# Crystal structure, Hirshfeld surface analysis and inter­action energy, DFT and anti­bacterial activity studies of ethyl 2-[(2*Z*)-2-(2-chloro­benzyl­idene)-3-oxo-3,4-di­hydro-2*H*-1,4-benzo­thia­zin-4-yl]acetate

**DOI:** 10.1107/S2056989020004119

**Published:** 2020-04-07

**Authors:** Ghizlane Sebbar, Ellouz Mohamed, Tuncer Hökelek, Joel T. Mague, Nada Kheira Sebbar, El Mokhtar Essassi, Bouchra Belkadi

**Affiliations:** a Laboratory of Microbiology and Molecular Biology, Faculty of Sciences, University Mohammed V, Rabat, Morocco; b Laboratoire de Chimie Organique Heterocyclique URAC 21, Pole de Competence Pharmacochimie, Faculté des Sciences, Université Mohammed V, Rabat, Morocco; c Department of Physics, Hacettepe University, 06800 Beytepe, Ankara, Turkey; d Department of Chemistry, Tulane University, New Orleans, LA 70118, USA; e Laboratoire de Chimie Appliquee et Environnement, Equipe de Chimie Bioorganique Appliquee, Faculte des Sciences, Université Ibn Zohr, Agadir, Morocco

**Keywords:** crystal structure, hydrogen bond, di­hydro­benzo­thia­zine, anti­bacterial activity, Hirshfeld surface

## Abstract

The title compound, C_19_H_16_ClNO_3_S, consists of chloro­phenyl methyl­idene and di­hydro­benzo­thia­zine units linked to an acetate moiety, where the thia­zine ring adopts a screw-boat conformation. In the crystal, two sets of weak C—H_Ph_⋯O_Dbt_ (Ph = phenyl and Dbt = di­hydro­benzo­thia­zine) hydrogen bonds form layers of mol­ecules parallel to the *bc* plane. The layers stack along the *a*-axis direction with inter­calation of the ester chains. The crystal studied was a two component twin with a refined BASF of 0.34961 (5). The Hirshfeld surface analysis of the crystal structure indicates that the most important contributions to the crystal packing are from H⋯H (37.5%), H⋯C/C⋯H (24.6%) and H⋯O/O⋯H (16.7%) inter­actions. Hydrogen-bonding and van der Waals inter­actions are the dominant inter­actions in the crystal packing. Computational chemistry indicates that in the crystal, C—H_Ph_⋯O_Dbt_ hydrogen bond energies are 38.3 and 30.3 kJ mol^−1^. Density functional theory (DFT) optimized structures at the B3LYP/ 6–311 G(d,p) level are compared with the experimentally determined mol­ecular structure in the solid state. The HOMO–LUMO behaviour was elucidated to determine the energy gap. Moreover, the anti­bacterial activity of the title compound has been evaluated against gram-positive and gram-negative bacteria.

## Abstract 2

The di­hydro­benzo­thia­zine ring is distinctly folded across the S⋯N axis and a puckering analysis of its conformation was performed. In the crystal, two sets of weak C—H_Ph_⋯O_Dbt_ (Ph = phenyl and Dbt = di­hydro­benzo­thia­zine) hydrogen bonds form layers parallel to the *bc* plane. The layers stack along the *a*-axis direction with inter­calation of the ester chains.

## Chemical context   

A number of pharmacological tests have revealed 1,4-benzo­thia­zine derivatives to possess a wide spectrum of biological applications, indicating that the 1,4-benzo­thia­zine moiety is a potentially useful template in medicinal chemistry research and therapeutic applications such as *in vivo* anti­proliferative (Zięba *et al.*, 2016 ▸), anti­bacterial (Sebbar *et al.*, 2016*b* ▸; Ellouz *et al.*, 2019 ▸), anti­microbial (Armenise *et al.*, 2012 ▸; Sabatini *et al.*, 2008 ▸; Vijay & Rahul, 2016 ▸), anti-viral (Malagu *et al.*, 1998 ▸), anti-oxidant (Zia-ur-Rehman *et al.*, 2009 ▸), anti-inflammatory (Trapani *et al.*, 1985 ▸; Gowda *et al.*, 2011 ▸), anti­pyretic (Warren & Knaus, 1987 ▸) and anti-cancer (Gupta & Gupta, 1991 ▸; Gupta *et al.*, 1985 ▸) areas. They have also been reported as precursors for the syntheses of new compounds (Sebbar *et al.*, 2015*a* ▸; Vidal *et al.*, 2006 ▸) possessing anti-diabetic (Tawada *et al.*, 1990 ▸) and anti-corrosion (Ellouz *et al.*, 2016*a* ▸,*b* ▸) activities, and as anti­proliferative (Zięba *et al.*, 2010 ▸) or anti­helmintic (Munirajasekar *et al.*, 2011 ▸) agents. The biological activities of some 1,4-benzo­thia­zines are similar to those of pheno­thia­zines, featuring the same structural specificity (Hni *et al.*, 2019*a* ▸,*b* ▸; Ellouz *et al.*, 2017*a* ▸, 2018 ▸, 2019 ▸; Sebbar *et al.*, 2019*a* ▸,*b* ▸). In a continuation of our research activities devoted to the development of N-substituted 1,4-benzo­thia­zine derivatives and the evaluation of their potential pharmacological activities (Ellouz *et al.*, 2017*a* ▸; Sebbar *et al.*, 2017*a* ▸), we have synthesized a new heterocyclic system containing 1,4-benzo­thia­zine. We report herein the synthesis and the mol­ecular and crystal structures along with the Hirshfeld surface analysis and inter­action energy calculations [using the CE–B3LYP/6–31G(d,p) energy model] and the density functional theory (DFT) computational calculations carried out at the B3LYP/6–311 G(d,p) level compared with the experimentally determined mol­ecular structure in the solid state. Moreover, the anti­bacterial activity of the title compound has been evaluated against gram-positive and gram-negative bacteria (*e.g. Staphylococcus aureus*, *Escherichia coli* and *Pseudomonas aeruginosa*).
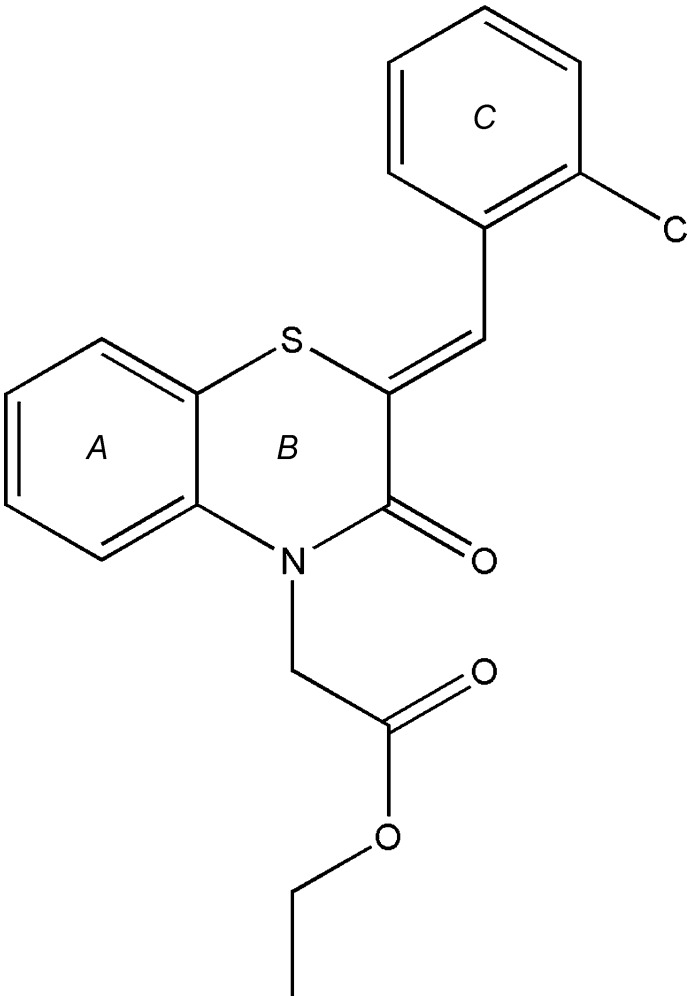


## Structural commentary   

**Figure 1:**
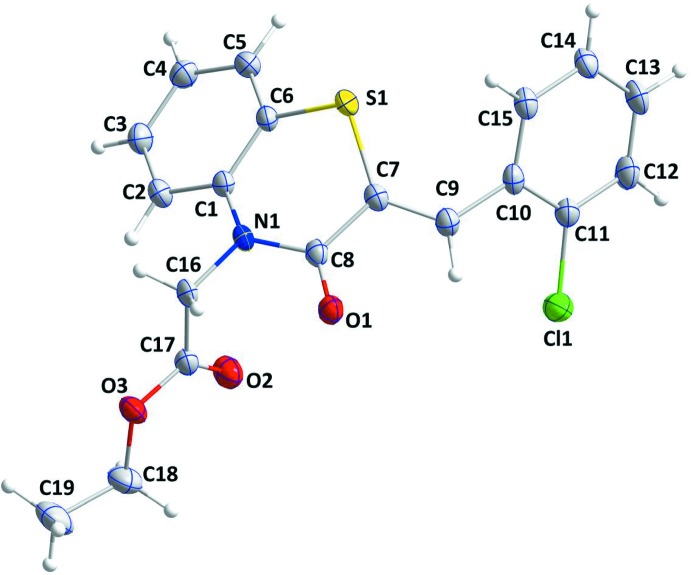
The asymmetric unit of the title compound with the atom-numbering scheme. Displacement ellipsoids are drawn at the 50% probability level.

The title compound, (I), consists of chloro­phenyl methyl­idene and di­hydro­benzo­thia­zine units linked to an acetate moiety, where the thia­zine ring adopts a screw-boat conformation (Fig. 1 ▸). The di­hydro­benzo­thia­zine ring is folded across the N1⋯S1 axis by 36.70 (7)°. A puckering analysis of the thia­zine, *B* (N1/S1/C1/C6–C8), ring conformation gave the parameters *Q*_T_ = 0.5525 (16) Å, θ = 109.0 (2)° and φ = 161.0 (2)°, indicating a screw-boat conformation. The mean plane of the N1/C16/C17/O2/O3 group is inclined to the mean plane of the S1/C1–C6/N1 unit by 80.06 (7)° while the phenyl, *C* (C10–C15), ring makes a dihedral angle of 84.92 (6)° with the latter plane. The benzene ring *A* (C1–C6) is oriented at a dihedral angle of 84.46 (2)° with respect to the *C* ring.

## Supra­molecular features   

**Figure 2:**
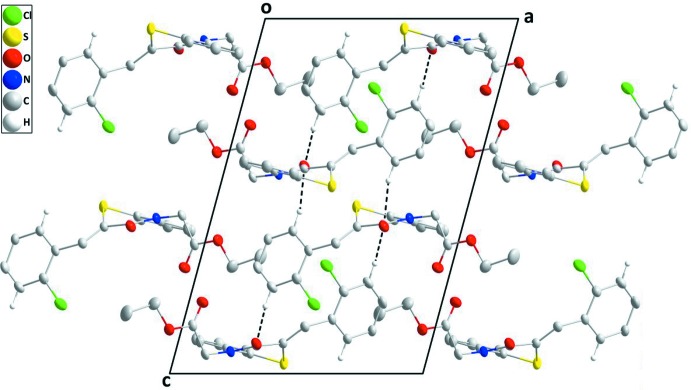
A partial packing diagram viewed along the *b*-axis direction. The weak C—H_Ph_⋯O_Dbt_ (Ph = phenyl and Dbt = di­hydro­benzo­thia­zine) hydrogen bonds are depicted by black dashed lines.

In the crystal, two sets of weak C—H_Ph_⋯O_Dbt_ (Ph = phenyl and Dbt = di­hydro­benzo­thia­zine) hydrogen bonds (Table 1 ▸) form layers of mol­ecules parallel to the *bc* plane (Fig. 2 ▸). The layers stack along the *a*-axis direction with inter­calation of the ester chains (Fig. 2 ▸).

## Hirshfeld surface analysis   

**Figure 3:**
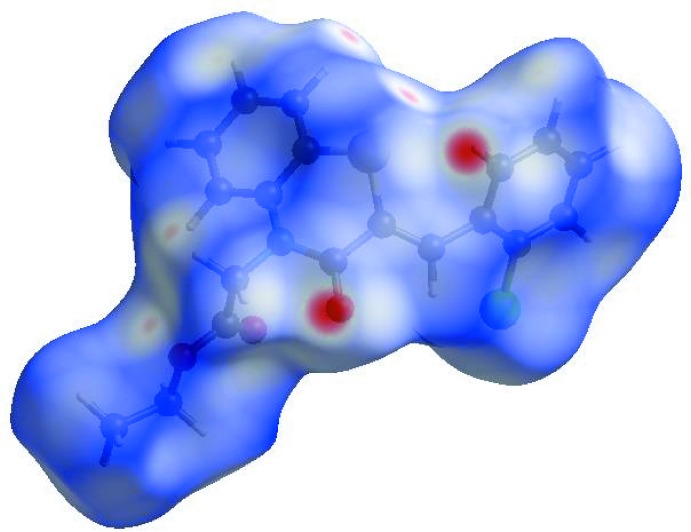
View of the three-dimensional Hirshfeld surface of the title compound plotted over *d*_norm_ in the range −0.1956 to 1.3971 a.u.

**Figure 4:**
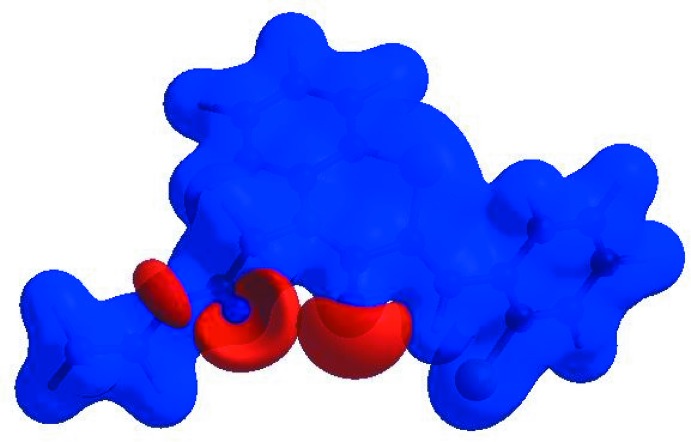
View of the three-dimensional Hirshfeld surface of the title compound plotted over electrostatic potential energy in the range −0.0500 to 0.0500 a.u. using the STO-3 G basis set at the Hartree–Fock level of theory. Hydrogen-bond donors and acceptors are shown as blue and red regions around the atoms corresponding to positive and negative potentials, respectively.

**Figure 5:**
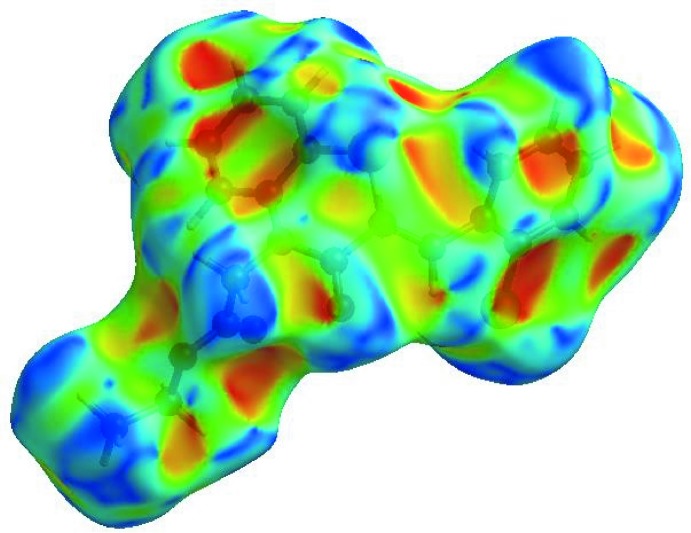
Hirshfeld surface of the title compound plotted over shape-index.

In order to visualize the inter­molecular inter­actions in the crystal of the title compound, a Hirshfeld surface (HS) analysis (Hirshfeld, 1977 ▸; Spackman & Jayatilaka, 2009 ▸) was carried out using *Crystal Explorer 17.5* (Turner *et al.*, 2017 ▸). In the HS plotted over *d*_norm_ (Fig. 3 ▸), the white surface indicates contacts with distances equal to the sum of van der Waals radii, and the red and blue colours indicate distances shorter (in close contact) or longer (distant contact) than the van der Waals radii, respectively (Venkatesan *et al.*, 2016 ▸). The bright-red spots appearing near O1 and hydrogen atom H15 indicate their roles as the respective donors and/or acceptors; they also appear as blue and red regions corresponding to positive and negative potentials on the HS mapped over electrostatic potential (Spackman *et al.*, 2008 ▸; Jayatilaka *et al.*, 2005 ▸) as shown in Fig. 4 ▸. Here the blue regions indicate positive electrostatic potential (hydrogen-bond donors), while the red regions indicate negative electrostatic potential (hydrogen-bond acceptors). The shape-index of the HS is a tool to visualize the π–π stacking by the presence of adjacent red and blue triangles; if there are no adjacent red and/or blue triangles, then there are no π–π inter­actions. Fig. 5 ▸ clearly suggests that there are no π–π inter­actions in (I).

**Figure 6:**
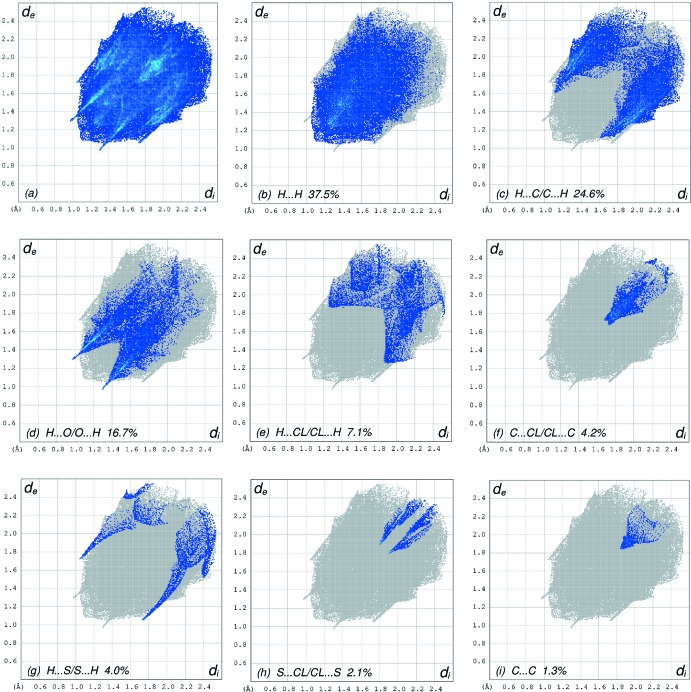
The full two-dimensional fingerprint plots for the title compound, showing (*a*) all inter­actions, and delineated into (*b*) H⋯H, (*c*) H⋯C/C⋯H, (*d*) H⋯O/O⋯H, (*e*) H⋯Cl/Cl ⋯ H, (*f*) C⋯Cl/Cl⋯C, (*g*) H⋯S/S⋯H, (*h*) S ⋯ Cl/Cl⋯S and (*i*) C⋯C inter­actions. The *d*_i_ and *d*_e_ values are the closest inter­nal and external distances (in Å) from given points on the Hirshfeld surface.

The overall two-dimensional fingerprint plot, Fig. 6 ▸ *a*, and those delineated into H⋯H, H⋯C/C⋯H, H⋯O/O⋯H, H⋯Cl/Cl⋯H, C⋯Cl/Cl⋯C, H⋯S/S⋯H, S⋯Cl/Cl⋯S and C⋯C contacts (McKinnon *et al.*, 2007 ▸) are illustrated in Fig. 6 ▸ *b*–*i*, respectively, together with their relative contributions to the Hirshfeld surface. The most important inter­action is H⋯H (Table 2 ▸) contributing 37.5% to the overall crystal packing, which is reflected in Fig. 6 ▸ *b* as widely scattered points of high density due to the large hydrogen-atom content of the mol­ecule with the tip at *d*_e_ = *d*_i_ = 1.10 Å. The pair of characteristic wings in the fingerprint plot delineated into H⋯C/C⋯H contacts (Table 2 ▸, Fig. 6 ▸ *c*; 24.6% contribution to the HS), have tips at *d*_e_ + *d*_i_ = 2.72 Å. The H⋯O/O⋯H contacts (Table 1 ▸, Fig. 6 ▸ *d*) with a 16.7% contribution to the HS have a symmetric distribution of points with the tips at *d*_e_ + *d*_i_ = 2.27 Å. The scattered points in the wings in the fingerprint plot delineated into H⋯Cl/Cl⋯H, Fig. 6 ▸ *e*, contacts (7.1% contribution) have the tips at *d*_e_ + *d*_i_ = 3.14 Å. The C⋯Cl/Cl⋯C contacts, Fig. 6 ▸ *f*, with 4.2% contribution to the HS have an arrow-shaped distribution of points of split small wings with the tips at *d*_e_ + *d*_i_ = 3.41 Å. The pair of spikes in the fingerprint plot delineated into H⋯S/S⋯H, Fig. 6 ▸ *g*, contacts (4.0% contribution) have tips at *d*_e_ + *d*_i_ = 2.78 Å. The pair of characteristic wings in the fingerprint plot delineated into S⋯Cl/Cl⋯S contacts, Fig. 6 ▸ *h*, (2.1% contribution) has the tips at *d*_e_ + *d*_i_ = 3.70 Å. Finally, the C⋯C contacts, Fig. 6 ▸ *i*, (1.3% contribution) have an arrow-shaped distribution of points with the tip at *d*_e_ = *d*_i_ = 1.85 Å.

**Figure 7:**
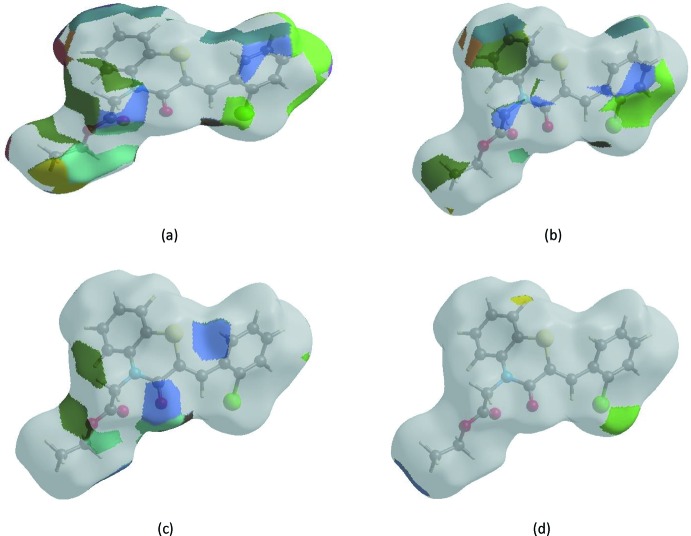
Hirshfeld surface representations with the function *d*_norm_ plotted onto the surface for (*a*) H⋯H, (*b*) H⋯C/C⋯H, (*c*) H⋯O/O⋯H and (*d*) H⋯Cl/Cl⋯H inter­actions.

The Hirshfeld surface representations with the function *d*_norm_ plotted onto the surface are shown for the H⋯H, H⋯C/C⋯H, H⋯O/O⋯H and H⋯Cl/Cl⋯H inter­actions in Fig. 7 ▸ *a*-*d*, respectively.

The Hirshfeld surface analysis confirms the importance of H-atom contacts in establishing the packing. The large number of H⋯H, H⋯C/C⋯H and H⋯O/O⋯H inter­actions suggest that van der Waals inter­actions and hydrogen bonding play the major roles in the crystal packing (Hathwar *et al.*, 2015 ▸).

## Inter­action energy calculations   

The inter­molecular inter­action energies were calculated using the CE–B3LYP/6–31G(d,p) energy model available in *Crystal Explorer 17.5* (Turner *et al.*, 2017 ▸), where a cluster of mol­ecules is generated by applying crystallographic symmetry operations with respect to a selected central mol­ecule within a default radius of 3.8 Å (Turner *et al.*, 2014 ▸). The total inter­molecular energy (*E*_tot_) is the sum of electrostatic (*E*_ele_), polarization (*E*_pol_), dispersion (*E*_dis_) and exchange-repulsion (*E*_rep_) energies (Turner *et al.*, 2015 ▸) with scale factors of 1.057, 0.740, 0.871 and 0.618, respectively (Mackenzie *et al.*, 2017 ▸). Hydrogen-bonding inter­action energies (in kJ mol^−1^) were calculated to be −20.3 (*E*_ele_), −5.9 (*E*_pol_), −48.7 (*E*_dis_), 48.5 (*E*_rep_) and −38.3 (*E*_tot_) for C15—H15⋯O1 and −15.2 (*E*_ele_), −4.1 (*E*_pol_), −42.2 (*E*_dis_), 41.3 (*E*_rep_) and −30.3 (*E*_tot_) for C12—H12⋯O1.

## DFT calculations   

**Figure 8:**
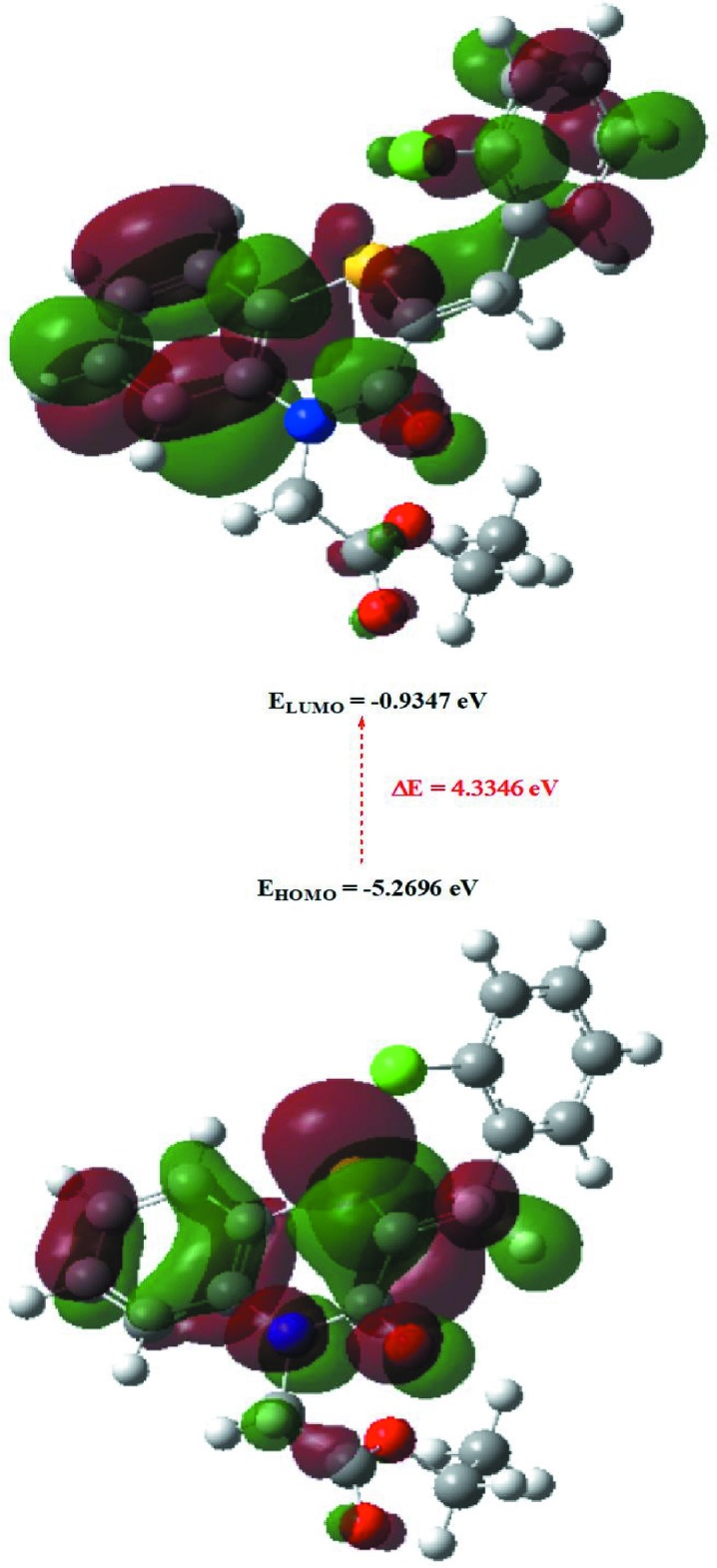
The energy band gap of the title compound, (I).

The optimized structure of the title compound, (I), in the gas phase was generated theoretically *via* density functional theory (DFT) using the standard B3LYP functional and 6–311 G(d,p) basis-set calculations (Becke, 1993 ▸) as implemented in *GAUSSIAN 09* (Frisch *et al.*, 2009 ▸). The theoretical and experimental results are in good agreement (Table 3 ▸). The highest-occupied mol­ecular orbital (HOMO), acting as an electron donor, and the lowest-unoccupied mol­ecular orbital (LUMO), acting as an electron acceptor, are important parameters for quantum chemistry. When the energy gap is small, the mol­ecule is highly polarizable and has high chemical reactivity. The DFT calculations provide some important information on the reactivity and site selectivity of the mol­ecular framework. *E*_HOMO_ and *E*_LUMO_ clarify the inevitable charge-exchange collaboration inside the studied material, electronegativity (χ), hardness (η), potential (μ), electrophilicity (ω) and softness (*σ*) are recorded in Table 4 ▸. The parameters η and *σ* are significant for the evaluation of both the reactivity and stability. The electron transition from the HOMO to the LUMO energy level is shown in Fig. 8 ▸. The HOMO and LUMO are localized in the plane extending from the whole 2-[(2*Z*)-2-(2-chloro­benzyl­idene)-3-oxo-3,4-di­hydro-2*H*-1,4-benzo­thia­zin-4-yl]acetate ring. The energy band gap [Δ*E* = *E*_LUMO_ - *E*_HOMO_] of the mol­ecule is 4.3346 eV, and the frontier mol­ecular orbital energies, *E*_HOMO_ and *E*_LUMO_ are −5.2696 and −0.9347 eV, respectively.

## Database survey   

A search of the Cambridge Structural Database (Version 5.38; Groom *et al.*, 2016 ▸) with the fragment (II) yielded 16 hits. The largest group is that for (III) with *R* = Ph and *R*′ = *A* [Chem scheme2] (WUFGIP; Sebbar *et al.*, 2015*b* ▸), CH_2_COOH (APAJUY; Sebbar *et al.*, 2016*a* ▸), (CH_2_)_17_CH_3_ (CARCEG; Sebbar *et al.*, 2017*a* ▸), *n*-Bu (JOGVOS; Sebbar *et al.*, 2014*a* ▸), CH_2_C≡CH (COGRUN; Sebbar *et al.*, 2014*b* ▸), *R* = Ph and *R*′ = *B* [Chem scheme2] (EVIYIT; (Sebbar *et al.*, 2016*b* ▸), CH_2_COOCH_3_ (ICAJOL; Zerzouf *et al.*, 2001 ▸), *R* = Ph and *R*′ = *C* [Chem scheme2] (JADPOW; Ellouz *et al.*, 2015 ▸) and *R* = Ph and *R*′ = *D* [Chem scheme2] (OBITUR; Sebbar *et al.*, 2016*c* ▸). The remainder have *R* = 4-ClC_6_H_4_ and *R*′ = bz (OMEGEU; Ellouz *et al.*, 2016*c* ▸), *n*-Bu (PAWCIC; Ellouz *et al.*, 2017*a* ▸) and *R* = 4-ClC_6_H_4_ and *R*′ = *B* [Chem scheme2] (YANHAZ; Ellouz *et al.*, 2017*b* ▸) or *R* = 2-ClC_6_H_4_, and *R*′ = CH_2_C≡CH (SAVTUH; Sebbar *et al.*, 2017*b* ▸) or *R* = 4-FC_6_H_4_ and *R*′ = CH_2_C≡CH (WOCFUS; Hni *et al.*, 2019*a* ▸) or *R* = 2,4-Cl_2_C_6_H_3_ and *R*′ = *B* (DOHZUY; Hni *et al.*, 2019*b* ▸, CH_2_CH_2_CN (POHPOU; Sebbar *et al.*, 2019*a* ▸). In the majority of these, the thia­zine ring is significantly folded about the S⋯N axis with dihedral angles between the two S/C/C/N planes ranging from *ca* 35° (JADPOW and WUFGIP) to *ca* 27° (COGRUN and WOCFUS). Two others have inter­mediate values of *ca* 15° (POHPOU) and 9° (DOHZUY), while in the last three, the thia­zine ring is nearly flat with a dihedral angle of *ca* 4° (EVIYIT, OBITUR and OMEGEU). It is not immediately obvious what the reasons are for these nearly planar rings, but it may be in part due to packing considerations since in these last three mol­ecules, the substituents on the thia­zine rings do not hold the benzo­thia­zine moieties as far apart as in the other cases, so that π-stacking inter­actions between the benzo portions can bring them close together and flatten out the rings.
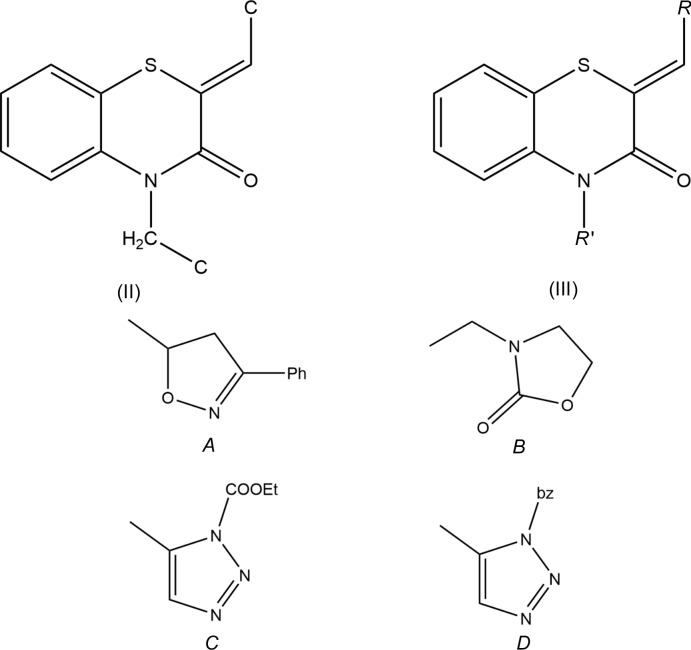


## Anti­bacterial activity   

**Figure 9:**
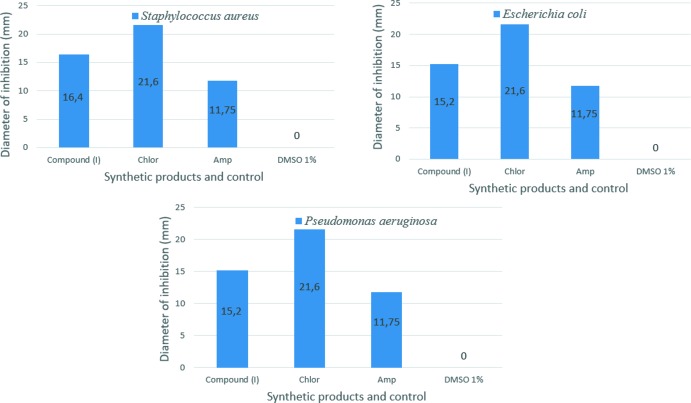
Anti­bacterial activity of the title compound, (I), and the commercial anti­biotics chloramphenicol (Chlor) and ampicillin (Amp) against the bacteria *Staphylococcus aureus*, *Escherichia coli* and *Pseudomonas aeruginosa*.

To compare and analyse the anti­bacterial behaviour contributed by (I), and commercial anti­biotics such as Chloramphenicol (Chlor) and Ampicillin (Amp), we have tested the title compound, (I), against *Staphylococcus aureus* (ATCC-25923), *Escherichia coli* (ATTC-25922) and *Pseudomonas aeruginosa* (ATCC-27853) strains of bacteria using the diffusion disk method to evaluate the applicability of (I) as an anti­bacterial agent (Mabkhot *et al.*, 2016 ▸; Hoffmann *et al.*, 2017 ▸). Fig. 9 ▸ summarizes the diameter of inhibition (mm) values of (I) and commercial anti­biotics chloramphenicol (Chlor) and ampicillin (Amp) against *Staphylococcus aureus*, *Escherichia coli* and *Pseudomonas aeruginosa*. The deter­min­ation of the minimum inhibition concentration (MIC) values of the sample (I) against the bacteria are presented in Table 5 ▸. The results of anti­bacterial activity of the product tested showed the best activity with MIC value of 21 µg mL^−1^ and different degrees of growth inhibition against the bacteria tested. It is clear that there is a significant enhancement and a strong anti­bacterial activity associated with sample (I), as compared to commercial anti­biotics. In addition, the maximum effect of (I) was recorded against *Staphylococcus aureus* (diameter of inhibition 16.4 mm). Chloramphenicol and ampicillin present a moderate anti­bacterial activity diameter of inhibition 22.6 mm and 11.75 mm, respectively, and no zone inhibition was observed with DMSO. On one hand, the chemical structure of (I) can explain this biologic effect. The mechanism of action of (I) is not attributable to one specific mechanism, but there are several targets in the cell: degradation of the cell wall, damage to membrane proteins, damage to cytoplasmic membrane, leakage of cell contents and coagulation of cytoplasm. On the other hand, it should be noted that the derivatives functionalized by ester groups and benzene rings have the highest anti­bacterial coefficient (92% of pathogenic bacteria are sensitive). This study is expected to include anti-inflammatory, anti­fungal, anti-parasitic and anti-cancer activities, because the literature gives a lot of inter­esting results on these topics. Some other types of bacteria may possibly be tested by employing the same method so as to eventually generalize the suggested investigation method (Alderman & Smith, 2001 ▸).

## Synthesis and crystallization   

To a solution of 2-(2-chloro­benzyl­idene)-3,4-di­hydro-2*H*-1,4-benzo­thia­zin-3-one (0.57 g, 2 mmol), potassium carbonate (4 mmol) and tetra *n*-butyl ammonium bromide (0.2 mmol) in DMF (14 ml) was added ethyl chloro­acetate (0.49 g, 4 mmol). Stirring was continued at room temperature for 14 h. The mixture was filtered and the solvent removed. The residue was extracted with water. The organic compound was chromatographed on a column of silica gel with ethyl acetate–hexane (8:2) as eluent. Colourless crystals of the title compound, (I), were isolated when the solvent was allowed to evaporate (yield: 66%).

## Refinement   

Crystal data, data collection and structure refinement details are summarized in Table 6 ▸. Hydrogen atoms were located in a difference-Fourier map and refined freely. The model was refined as a two-component twin with twin law 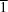 0 0, 0 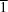 0, 0 0 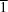 and a refined BASF parameter of 0.34961 (5).

## Supplementary Material

Crystal structure: contains datablock(s) I, global. DOI: 10.1107/S2056989020004119/lh5950sup1.cif

Structure factors: contains datablock(s) I. DOI: 10.1107/S2056989020004119/lh5950Isup2.hkl

Supporting information file. DOI: 10.1107/S2056989020004119/lh5950Isup3.cdx

Supporting information file. DOI: 10.1107/S2056989020004119/lh5950Isup4.cml

CCDC reference: 1992626

Additional supporting information:  crystallographic information; 3D view; checkCIF report

## Figures and Tables

**Table 1:** Hydrogen-bond geometry (Å, °)

*D*—H⋯*A*	*D*—H	H⋯*A*	*D*⋯*A*	*D*—H⋯*A*
C12—H12⋯O1^vii^	0.95 (3)	2.56 (3)	3.214 (2)	126 (2)
C15—H15⋯O1^ii^	0.95 (2)	2.40 (2)	3.227 (2)	145.8 (15)

Symmetry codes: (ii) 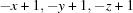; (vii) 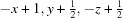.

**Table 2:** Selected interatomic distances (Å)

Cl1⋯C14^i^	3.5363 (9)	O1⋯H12^i^	2.560 (9)
Cl1⋯S1^i^	3.7268 (3)	O1⋯H15^ii^	2.400 (8)
Cl1⋯C10^i^	3.5940 (7)	O2⋯H18*B*	2.545 (11)
Cl1⋯C15^i^	3.4359 (7)	O2⋯H13^i^	2.606 (10)
Cl1⋯H9	2.674 (8)	O2⋯H18*A*	2.74 (3)
S1⋯N1	3.0100 (6)	O3⋯H16*B* ^vi^	2.666 (8)
S1⋯C15	3.1748 (8)	C2⋯C17	3.2932 (10)
S1⋯O1^ii^	3.4189 (6)	C4⋯C14^iii^	3.5882 (12)
S1⋯H15	2.660 (10)	C2⋯H16*B*	2.621 (8)
S1⋯H5^iii^	2.906 (9)	C4⋯H14^iii^	2.826 (10)
O1⋯C17	3.1263 (9)	C4⋯H12^vii^	2.993 (10)
O1⋯C12^i^	3.2141 (10)	C5⋯H12^vii^	2.817 (10)
O1⋯C15^ii^	3.2268 (8)	C7⋯H15	2.937 (9)
O2⋯N1	2.7902 (7)	C15⋯H16*A* ^ii^	2.900 (9)
O2⋯C8	3.2091 (9)	C16⋯H16*B* ^vi^	2.987 (9)
O2⋯C1	3.3715 (8)	C16⋯H2	2.538 (9)
O2⋯C3^iv^	3.3498 (10)	C17⋯H2	2.696 (9)
O2⋯C2	3.4103 (9)	H2⋯H16*B*	2.223 (12)
O1⋯H9	2.516 (9)	H5⋯H12^vii^	2.444 (13)
O1⋯H16*A*	2.376 (9)	H5⋯H15^iii^	2.509 (12)
O1⋯H4^v^	2.806 (11)	H16*B*⋯H16*B* ^vi^	2.381 (13)

Symmetry codes: (i) 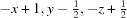; (ii) 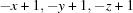; (iii) 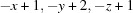; (iv) 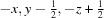; (v) 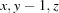; (vi) 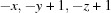; (vii) 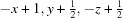.

**Table 3:** Comparison of selected (X-ray and DFT) geometric data (Å, °)

Bonds/angles	X-ray	B3LYP/6–311G(d,p)
Cl1—C11	1.741 (2)	1.83593
S1—C6	1.755 (2)	1.83362
S1—C7	1.757 (2)	1.79349
O1—C8	1.224 (2)	1.26839
O2—C17	1.200 (2)	1.23993
O3—C17	1.335 (2)	1.36867
O3—C18	1.462 (3)	1.48321
N1—C8	1.381 (2)	1.40044
N1—C1	1.417 (2)	1.41683
N1—C16	1.452 (2)	1.47008
		
C6—S1—C7	98.19 (9)	99.41730
C17—O3—C18	116.60 (16)	116.97676
C8—N1—C1	124.52 (15)	125.49531
C8—N1—C16	115.56 (16)	115.02066
C1—N1—C16	118.47 (16)	118.38057
C2—C1—N1	121.41 (17)	121.23845
C2—C1—C6	118.60 (18)	117.94010
C6—C1—N1	120.00 (17)	120.81444
O1—C8—N1	120.36 (17)	120.12402
O1—C8—C7	121.99 (17)	120.12402
N1—C8—C7	117.64 (16)	117.79908

**Table 4:** Calculated energies

Mol­ecular Energy (a.u.) (eV)	Compound (I)
Total Energy, *TE* (eV)	−50964
E_HOMO_ (eV)	−5.2696
E_LUMO_ (eV)	−0.9347
Gap, *ΔE* (eV)	4.3346
Dipole moment, *μ* (Debye)	5.6841
Ionization potential, *I* (eV)	5.2696
Electron affinity, *A*	0.9347
Electronegativity, *χ*	3.1019
Hardness, *η*	2.1673
Electrophilicity index, *ω*	2.2198
Softness, *σ*	0.4614
Fraction of electron transferred, *ΔN*	0.8993

**Table 5:** Minimal inhibitory concentration [MIC (μg m*L* ^−1^)] ATCC-25923 = *Staphylococcus aureus*, ATTC-25922 = *Escherichia coli*, ATCC-27853 = *Pseudomonas aeruginosa*, Chlor = chloramphenicol and Amp = ampicillin.

Product	ATCC-25923	ATTC-25922	ATCC-27853
(I)	21	21	21
Chlor	58	58	58
Amp	12	12	12
DMSO	0	0	0

**Table 6:** Experimental details

Crystal data	
Chemical formula	C_19_H_16_ClNO_3_S
*M*_r_	373.84
Crystal system, space group	Monoclinic, *P*2_1_/*c*
Temperature (K)	150
*a*, *b*, *c* (Å)	11.6882 (2), 9.0903 (2), 16.9533 (3)
β (°)	105.105 (1)
*V* (Å^3^)	1739.04 (6)
*Z*	4
Radiation type	Cu *K*α
μ (mm^−1^)	3.22
Crystal size (mm)	0.19 × 0.15 × 0.11

Data collection	
Diffractometer	Bruker D8 VENTURE PHOTON 100 CMOS
Absorption correction	Multi-scan (*TWINABS*; Sheldrick, 2009 ▸)
*T*_min_, *T*_max_	0.57, 0.72
No. of measured, independent and observed [*I* > 2σ(*I*)] reflections	25761, 25761, 21950
*R*_int_	0.032
(sin θ/λ)_max_ (Å^−1^)	0.625

Refinement	
*R*[*F* ^2^ > 2σ(*F* ^2^)], *wR*(*F* ^2^), *S*	0.039, 0.101, 1.03
No. of reflections	25761
No. of parameters	292
H-atom treatment	All H-atom parameters refined
Δρ_max_, Δρ_min_ (e Å^−3^)	0.72, −0.80

Computer programs: *APEX3* and *SAINT* (Bruker, 2016 ▸), *CELL_NOW* (Sheldrick, 2008*a* ▸), *SHELXT* (Sheldrick, 2015*a* ▸), *SHELXL2018* (Sheldrick, 2015*b* ▸), *DIAMOND* (Brandenburg & Putz, 2012 ▸) and *SHELXTL* (Sheldrick, 2008*b* ▸).

## supplementary crystallographic information

### Crystal data

**Table:**

C_19_H_16_ClNO_3_S	*F*(000) = 776
*M**_r_* = 373.84	*D*_x_ = 1.428 Mg m^−^^3^
Monoclinic, *P*2_1_/*c*	Cu *K*α radiation, λ = 1.54178 Å
*a* = 11.6882 (2) Å	Cell parameters from 9820 reflections
*b* = 9.0903 (2) Å	θ = 3.9–74.6°
*c* = 16.9533 (3) Å	µ = 3.22 mm^−^^1^
β = 105.105 (1)°	*T* = 150 K
*V* = 1739.04 (6) Å^3^	Block, colourless
*Z* = 4	0.19 × 0.15 × 0.11 mm

### Data collection

**Table:**

Bruker D8 VENTURE PHOTON 100 CMOS diffractometer	25761 independent reflections
Radiation source: INCOATEC IµS micro–focus source	21950 reflections with *I* > 2σ(*I*)
Mirror monochromator	*R*_int_ = 0.032
Detector resolution: 10.4167 pixels mm^-1^	θ_max_ = 74.6°, θ_min_ = 3.9°
ω scans	*h* = −14→13
Absorption correction: multi-scan (*TWINABS*; Sheldrick, 2009)	*k* = −11→10
*T*_min_ = 0.57, *T*_max_ = 0.72	*l* = −21→21
25761 measured reflections	

### Refinement

**Table:**

Refinement on *F*^2^	Secondary atom site location: difference Fourier map
Least-squares matrix: full	Hydrogen site location: difference Fourier map
*R*[*F*^2^ > 2σ(*F*^2^)] = 0.039	All H-atom parameters refined
*wR*(*F*^2^) = 0.101	*w* = 1/[σ^2^(*F*_o_^2^) + (0.0405*P*)^2^ + 0.4043*P*] where *P* = (*F*_o_^2^ + 2*F*_c_^2^)/3
*S* = 1.02	(Δ/σ)_max_ < 0.001
25761 reflections	Δρ_max_ = 0.72 e Å^−^^3^
292 parameters	Δρ_min_ = −0.80 e Å^−^^3^
0 restraints	Extinction correction: *SHELXL2018/1* (Sheldrick, 2015*b*), Fc^*^=kFc[1+0.001xFc^2^λ^3^/sin(2θ)]^-1/4^
Primary atom site location: dual space	Extinction coefficient: 0.0032 (6)

### Special details

**Table:**

Experimental. Analysis of 529 reflections having I/σ(I) > 12 and chosen from the full data set with *CELL_NOW* (Sheldrick, 2008a) showed the crystal to belong to the monoclinic system and to be twinned by a 180° rotation about the *b* axis. The raw data were processed using the multi- component version of *SAINT* under control of the two-component orientation file generated by *CELL_NOW*.
Geometry. All esds (except the esd in the dihedral angle between two l.s. planes) are estimated using the full covariance matrix. The cell esds are taken into account individually in the estimation of esds in distances, angles and torsion angles; correlations between esds in cell parameters are only used when they are defined by crystal symmetry. An approximate (isotropic) treatment of cell esds is used for estimating esds involving l.s. planes.
Refinement. Refinement of F^2^ against ALL reflections. The weighted R-factor wR and goodness of fit S are based on F^2^, conventional R-factors R are based on F, with F set to zero for negative F^2^. The threshold expression of F^2^ > 2sigma(F^2^) is used only for calculating R-factors(gt) etc. and is not relevant to the choice of reflections for refinement. R-factors based on F^2^ are statistically about twice as large as those based on F, and R- factors based on ALL data will be even larger. Refined as a 2-component twin.

### Fractional atomic coordinates and isotropic or equivalent isotropic displacement parameters (Å^2^)

**Table:**

	*x*	*y*	*z*	*U*_iso_*/*U*_eq_	
Cl1	0.50488 (5)	0.51497 (7)	0.19568 (3)	0.03952 (18)	
S1	0.44083 (4)	0.78070 (6)	0.47131 (3)	0.02735 (16)	
O1	0.31103 (13)	0.39288 (15)	0.41536 (8)	0.0264 (3)	
O2	0.05392 (14)	0.49512 (17)	0.29883 (9)	0.0341 (4)	
O3	−0.04896 (13)	0.37965 (16)	0.37586 (9)	0.0306 (3)	
N1	0.21745 (14)	0.60019 (17)	0.43805 (10)	0.0223 (3)	
C1	0.19875 (18)	0.7541 (2)	0.42964 (11)	0.0216 (4)	
C2	0.08488 (19)	0.8137 (2)	0.40863 (13)	0.0272 (4)	
H2	0.019 (2)	0.750 (3)	0.3964 (15)	0.032 (6)*	
C3	0.0683 (2)	0.9646 (2)	0.40245 (13)	0.0299 (5)	
H3	−0.010 (3)	1.002 (3)	0.3866 (16)	0.038 (7)*	
C4	0.1643 (2)	1.0587 (2)	0.41608 (13)	0.0294 (5)	
H4	0.152 (3)	1.159 (3)	0.4110 (17)	0.045 (7)*	
C5	0.2778 (2)	1.0009 (2)	0.43568 (13)	0.0274 (4)	
H5	0.345 (2)	1.066 (3)	0.4429 (15)	0.033 (6)*	
C6	0.29556 (17)	0.8496 (2)	0.44327 (11)	0.0228 (4)	
C7	0.41573 (18)	0.6168 (2)	0.41447 (11)	0.0224 (4)	
C8	0.31254 (18)	0.5270 (2)	0.42214 (11)	0.0214 (4)	
C9	0.48358 (18)	0.5650 (2)	0.36782 (12)	0.0239 (4)	
H9	0.461 (2)	0.474 (3)	0.3411 (15)	0.030 (6)*	
C10	0.58341 (18)	0.6384 (2)	0.34694 (12)	0.0230 (4)	
C11	0.59934 (19)	0.6260 (2)	0.26787 (12)	0.0266 (4)	
C12	0.6869 (2)	0.7022 (2)	0.24392 (13)	0.0308 (5)	
H12	0.693 (2)	0.689 (3)	0.1896 (16)	0.039 (7)*	
C13	0.7644 (2)	0.7914 (2)	0.29922 (13)	0.0316 (5)	
H13	0.826 (3)	0.842 (3)	0.2818 (17)	0.042 (7)*	
C14	0.75389 (19)	0.8019 (2)	0.37885 (13)	0.0281 (4)	
H14	0.808 (2)	0.863 (3)	0.4187 (16)	0.037 (7)*	
C15	0.66479 (18)	0.7271 (2)	0.40190 (12)	0.0255 (4)	
H15	0.659 (2)	0.733 (2)	0.4564 (15)	0.027 (6)*	
C16	0.11979 (18)	0.5077 (2)	0.44607 (12)	0.0235 (4)	
H16A	0.151 (2)	0.421 (3)	0.4763 (15)	0.029 (6)*	
H16B	0.077 (2)	0.556 (3)	0.4769 (14)	0.026 (6)*	
C17	0.03970 (18)	0.4622 (2)	0.36420 (12)	0.0244 (4)	
C18	−0.1348 (2)	0.3263 (3)	0.30254 (15)	0.0386 (5)	
H18A	−0.092 (3)	0.255 (3)	0.2763 (18)	0.048 (8)*	
H18B	−0.161 (3)	0.410 (3)	0.2641 (19)	0.049 (8)*	
C19	−0.2350 (3)	0.2585 (4)	0.3297 (2)	0.0509 (7)	
H19A	−0.205 (3)	0.173 (3)	0.370 (2)	0.054 (8)*	
H19B	−0.289 (3)	0.220 (4)	0.283 (2)	0.069 (10)*	
H19C	−0.274 (3)	0.331 (4)	0.359 (2)	0.061 (9)*	

### Atomic displacement parameters (Å^2^)

**Table:**

	*U*^11^	*U*^22^	*U*^33^	*U*^12^	*U*^13^	*U*^23^
Cl1	0.0417 (4)	0.0478 (3)	0.0343 (3)	−0.0153 (2)	0.0194 (2)	−0.0140 (2)
S1	0.0165 (3)	0.0318 (3)	0.0341 (3)	−0.00303 (19)	0.0072 (2)	−0.0083 (2)
O1	0.0279 (8)	0.0251 (7)	0.0300 (7)	−0.0012 (6)	0.0143 (6)	0.0008 (5)
O2	0.0335 (9)	0.0442 (9)	0.0270 (7)	−0.0083 (7)	0.0122 (7)	0.0003 (6)
O3	0.0253 (8)	0.0361 (8)	0.0318 (7)	−0.0111 (6)	0.0100 (6)	−0.0034 (6)
N1	0.0180 (8)	0.0251 (8)	0.0265 (8)	−0.0032 (6)	0.0104 (7)	0.0002 (6)
C1	0.0196 (10)	0.0247 (9)	0.0226 (8)	−0.0025 (8)	0.0093 (8)	−0.0020 (7)
C2	0.0178 (10)	0.0308 (10)	0.0340 (10)	−0.0033 (8)	0.0086 (8)	−0.0031 (8)
C3	0.0215 (11)	0.0327 (11)	0.0357 (11)	0.0027 (9)	0.0080 (9)	−0.0027 (9)
C4	0.0296 (12)	0.0246 (10)	0.0357 (11)	0.0008 (9)	0.0117 (9)	−0.0024 (8)
C5	0.0238 (11)	0.0282 (10)	0.0323 (10)	−0.0061 (8)	0.0108 (9)	−0.0064 (8)
C6	0.0170 (10)	0.0287 (10)	0.0241 (9)	−0.0019 (8)	0.0083 (8)	−0.0036 (7)
C7	0.0190 (10)	0.0253 (9)	0.0236 (9)	−0.0012 (7)	0.0072 (8)	0.0016 (7)
C8	0.0200 (10)	0.0274 (10)	0.0184 (8)	−0.0010 (8)	0.0076 (7)	0.0006 (7)
C9	0.0217 (10)	0.0254 (10)	0.0262 (9)	−0.0004 (8)	0.0094 (8)	0.0020 (8)
C10	0.0191 (10)	0.0252 (9)	0.0275 (9)	0.0040 (7)	0.0111 (8)	0.0039 (7)
C11	0.0243 (11)	0.0282 (10)	0.0300 (10)	0.0001 (8)	0.0121 (9)	−0.0012 (8)
C12	0.0313 (12)	0.0368 (12)	0.0298 (10)	−0.0013 (9)	0.0177 (9)	0.0006 (9)
C13	0.0249 (11)	0.0386 (12)	0.0359 (11)	−0.0044 (9)	0.0164 (9)	0.0038 (9)
C14	0.0181 (10)	0.0374 (11)	0.0296 (10)	−0.0027 (9)	0.0072 (8)	0.0006 (9)
C15	0.0196 (10)	0.0333 (10)	0.0248 (9)	0.0022 (8)	0.0083 (8)	0.0038 (8)
C16	0.0208 (10)	0.0267 (10)	0.0268 (9)	−0.0039 (8)	0.0129 (8)	0.0003 (8)
C17	0.0212 (10)	0.0239 (9)	0.0307 (10)	−0.0017 (8)	0.0114 (8)	−0.0017 (8)
C18	0.0306 (13)	0.0435 (13)	0.0387 (12)	−0.0113 (11)	0.0033 (10)	−0.0085 (11)
C19	0.0319 (15)	0.0561 (17)	0.0614 (17)	−0.0187 (13)	0.0065 (14)	−0.0055 (15)

### Geometric parameters (Å, º)

**Table:**

Cl1—C11	1.741 (2)	C9—C10	1.465 (3)
S1—C6	1.755 (2)	C9—H9	0.95 (2)
S1—C7	1.757 (2)	C10—C15	1.401 (3)
O1—C8	1.224 (2)	C10—C11	1.405 (3)
O2—C17	1.200 (2)	C11—C12	1.382 (3)
O3—C17	1.335 (2)	C12—C13	1.383 (3)
O3—C18	1.462 (3)	C12—H12	0.95 (3)
N1—C8	1.381 (2)	C13—C14	1.390 (3)
N1—C1	1.417 (2)	C13—H13	0.96 (3)
N1—C16	1.452 (2)	C14—C15	1.382 (3)
C1—C2	1.395 (3)	C14—H14	0.97 (3)
C1—C6	1.397 (3)	C15—H15	0.95 (2)
C2—C3	1.386 (3)	C16—C17	1.515 (3)
C2—H2	0.95 (3)	C16—H16A	0.96 (3)
C3—C4	1.382 (3)	C16—H16B	0.92 (3)
C3—H3	0.94 (3)	C18—C19	1.498 (4)
C4—C5	1.384 (3)	C18—H18A	0.99 (3)
C4—H4	0.93 (3)	C18—H18B	1.00 (3)
C5—C6	1.392 (3)	C19—H19A	1.03 (3)
C5—H5	0.96 (3)	C19—H19B	0.94 (4)
C7—C9	1.343 (3)	C19—H19C	1.00 (4)
C7—C8	1.490 (3)		
			
Cl1···C14^i^	3.5363 (9)	O1···H12^i^	2.560 (9)
Cl1···S1^i^	3.7268 (3)	O1···H15^ii^	2.400 (8)
Cl1···C10^i^	3.5940 (7)	O2···H18B	2.545 (11)
Cl1···C15^i^	3.4359 (7)	O2···H13^i^	2.606 (10)
Cl1···H9	2.674 (8)	O2···H18A	2.74 (3)
S1···N1	3.0100 (6)	O3···H16B^vi^	2.666 (8)
S1···C15	3.1748 (8)	C2···C17	3.2932 (10)
S1···O1^ii^	3.4189 (6)	C4···C14^iii^	3.5882 (12)
S1···H15	2.660 (10)	C2···H16B	2.621 (8)
S1···H5^iii^	2.906 (9)	C4···H14^iii^	2.826 (10)
O1···C17	3.1263 (9)	C4···H12^vii^	2.993 (10)
O1···C12^i^	3.2141 (10)	C5···H12^vii^	2.817 (10)
O1···C15^ii^	3.2268 (8)	C7···H15	2.937 (9)
O2···N1	2.7902 (7)	C15···H16A^ii^	2.900 (9)
O2···C8	3.2091 (9)	C16···H16B^vi^	2.987 (9)
O2···C1	3.3715 (8)	C16···H2	2.538 (9)
O2···C3^iv^	3.3498 (10)	C17···H2	2.696 (9)
O2···C2	3.4103 (9)	H2···H16B	2.223 (12)
O1···H9	2.516 (9)	H5···H12^vii^	2.444 (13)
O1···H16A	2.376 (9)	H5···H15^iii^	2.509 (12)
O1···H4^v^	2.806 (11)	H16B···H16B^vi^	2.381 (13)
			
C6—S1—C7	98.19 (9)	C12—C11—Cl1	117.82 (16)
C17—O3—C18	116.60 (16)	C10—C11—Cl1	119.98 (16)
C8—N1—C1	124.52 (15)	C11—C12—C13	119.92 (19)
C8—N1—C16	115.56 (16)	C11—C12—H12	118.0 (16)
C1—N1—C16	118.47 (16)	C13—C12—H12	122.0 (16)
C2—C1—C6	118.60 (18)	C12—C13—C14	119.4 (2)
C2—C1—N1	121.41 (17)	C12—C13—H13	118.8 (16)
C6—C1—N1	120.00 (17)	C14—C13—H13	121.8 (16)
C3—C2—C1	120.63 (19)	C15—C14—C13	120.3 (2)
C3—C2—H2	120.0 (15)	C15—C14—H14	119.2 (16)
C1—C2—H2	119.4 (15)	C13—C14—H14	120.5 (16)
C4—C3—C2	120.6 (2)	C14—C15—C10	121.74 (18)
C4—C3—H3	120.2 (16)	C14—C15—H15	120.3 (15)
C2—C3—H3	119.2 (16)	C10—C15—H15	118.0 (15)
C3—C4—C5	119.4 (2)	N1—C16—C17	112.65 (16)
C3—C4—H4	120.0 (18)	N1—C16—H16A	109.1 (15)
C5—C4—H4	120.6 (18)	C17—C16—H16A	109.0 (14)
C4—C5—C6	120.5 (2)	N1—C16—H16B	109.1 (15)
C4—C5—H5	119.4 (15)	C17—C16—H16B	110.8 (15)
C6—C5—H5	120.1 (15)	H16A—C16—H16B	106 (2)
C5—C6—C1	120.27 (18)	O2—C17—O3	125.18 (19)
C5—C6—S1	119.21 (15)	O2—C17—C16	125.21 (18)
C1—C6—S1	120.51 (15)	O3—C17—C16	109.61 (16)
C9—C7—C8	118.34 (17)	O3—C18—C19	107.0 (2)
C9—C7—S1	125.50 (16)	O3—C18—H18A	106.6 (17)
C8—C7—S1	116.11 (14)	C19—C18—H18A	113.0 (17)
O1—C8—N1	120.36 (17)	O3—C18—H18B	109.1 (17)
O1—C8—C7	121.99 (17)	C19—C18—H18B	112.6 (18)
N1—C8—C7	117.64 (16)	H18A—C18—H18B	108 (2)
C7—C9—C10	127.75 (19)	C18—C19—H19A	111.0 (18)
C7—C9—H9	116.7 (15)	C18—C19—H19B	108 (2)
C10—C9—H9	115.3 (15)	H19A—C19—H19B	109 (3)
C15—C10—C11	116.42 (18)	C18—C19—H19C	112.1 (19)
C15—C10—C9	123.16 (17)	H19A—C19—H19C	107 (2)
C11—C10—C9	120.39 (18)	H19B—C19—H19C	111 (3)
C12—C11—C10	122.19 (19)		
			
C8—N1—C1—C2	149.38 (19)	C9—C7—C8—N1	−152.68 (18)
C16—N1—C1—C2	−16.2 (3)	S1—C7—C8—N1	29.5 (2)
C8—N1—C1—C6	−31.3 (3)	C8—C7—C9—C10	175.30 (18)
C16—N1—C1—C6	163.13 (17)	S1—C7—C9—C10	−7.1 (3)
C6—C1—C2—C3	−0.8 (3)	C7—C9—C10—C15	37.4 (3)
N1—C1—C2—C3	178.55 (18)	C7—C9—C10—C11	−140.4 (2)
C1—C2—C3—C4	0.8 (3)	C15—C10—C11—C12	−2.9 (3)
C2—C3—C4—C5	0.3 (3)	C9—C10—C11—C12	175.1 (2)
C3—C4—C5—C6	−1.3 (3)	C15—C10—C11—Cl1	178.57 (15)
C4—C5—C6—C1	1.2 (3)	C9—C10—C11—Cl1	−3.5 (3)
C4—C5—C6—S1	−178.03 (16)	C10—C11—C12—C13	1.7 (3)
C2—C1—C6—C5	−0.2 (3)	Cl1—C11—C12—C13	−179.76 (18)
N1—C1—C6—C5	−179.53 (17)	C11—C12—C13—C14	0.7 (3)
C2—C1—C6—S1	179.07 (15)	C12—C13—C14—C15	−1.8 (3)
N1—C1—C6—S1	−0.3 (2)	C13—C14—C15—C10	0.5 (3)
C7—S1—C6—C5	−146.23 (16)	C11—C10—C15—C14	1.8 (3)
C7—S1—C6—C1	34.53 (17)	C9—C10—C15—C14	−176.10 (19)
C6—S1—C7—C9	134.12 (18)	C8—N1—C16—C17	−80.3 (2)
C6—S1—C7—C8	−48.28 (16)	C1—N1—C16—C17	86.5 (2)
C1—N1—C8—O1	−165.71 (18)	C18—O3—C17—O2	−0.1 (3)
C16—N1—C8—O1	0.3 (3)	C18—O3—C17—C16	−179.95 (18)
C1—N1—C8—C7	14.8 (3)	N1—C16—C17—O2	1.2 (3)
C16—N1—C8—C7	−179.20 (16)	N1—C16—C17—O3	−179.02 (16)
C9—C7—C8—O1	27.9 (3)	C17—O3—C18—C19	−171.0 (2)
S1—C7—C8—O1	−149.91 (15)		

Symmetry codes: (i) −*x*+1, *y*−1/2, −*z*+1/2; (ii) −*x*+1, −*y*+1, −*z*+1; (iii) −*x*+1, −*y*+2, −*z*+1; (iv) −*x*, *y*−1/2, −*z*+1/2; (v) *x*, *y*−1, *z*; (vi) −*x*, −*y*+1, −*z*+1; (vii) −*x*+1, *y*+1/2, −*z*+1/2.

### Hydrogen-bond geometry (Å, º)

**Table:**

*D*—H···*A*	*D*—H	H···*A*	*D*···*A*	*D*—H···*A*
C12—H12···O1^vii^	0.95 (3)	2.56 (3)	3.214 (2)	126 (2)
C15—H15···O1^ii^	0.95 (2)	2.40 (2)	3.227 (2)	145.8 (15)

Symmetry codes: (ii) −*x*+1, −*y*+1, −*z*+1; (vii) −*x*+1, *y*+1/2, −*z*+1/2.
